# Towards a new perspective: Exploring the variability of conditional risk factors for multimorbidity susceptibility among older adults in India

**DOI:** 10.1371/journal.pone.0323890

**Published:** 2025-06-17

**Authors:** Ajay Kumar, Suryakant Yadav

**Affiliations:** Department of Biostatistics and Epidemiology, International Institute for Population Sciences (IIPS), Mumbai, India; Iran University of Medical Sciences, IRAN, ISLAMIC REPUBLIC OF

## Abstract

**Background:**

There is a lack of substantive evidence on the epidemiology of multimorbidity in low and middle-income countries (LMICs). India is also undergoing through the major demographic shift marked by the rapidly aging population and followed by significantly elevated implications to multimorbidity risk. These implications include an increased risk of functional limitations, poor quality of life, and delayed mortality pattern but expanding morbidity burden among older adults. This study aimed to characterize the distribution, pattern and further provide an understanding of the conditional role of risk factors for multimorbidity susceptibility among older adults in India.

**Methods:**

The study utilized data on 66,606 individuals form the national representative Longitudinal Ageing Study in India (LASI), Wave – 1, of individuals aged 45 years and above. To understands the distribution, prevalence and pattern of morbidities and multimorbidity we characterized and calculated the weighted frequency distribution, chi-square test for association and stacked area plot for relative burden of multimorbidity over age. Finally, we constructed the Classification and Regression Trees (CART) model to further identify the optimal leading covariates and their conditional role on multimorbidity susceptibility among older adults in India.

**Results:**

Hypertension showed the highest prevalence (26.72%) and highest age-specific relative proportional share, followed by myopia (24.2%), hypermetropia (20.75%) and gastrointestinal condition (17.98%). Single morbidity prevalence was 23.78%, and multimorbidity was 50.94% and the relative prevalence share of multimorbidity over age expanded by 25.16% from the 45–49 to 85+ age group. The CART model identified that childhood health, place of residence, age, body mass index, caste category and education level were the leading risk factors for multimorbidity susceptibility among older adults in India. The highest susceptibility of multimorbidity with a risk of 0.57 was observed among individuals who had moderate to poor childhood health. Conversely, those adults who had good childhood health, resided in rural areas, underweighted or normal BMI and belonged to the Schedule tribe had the lowest risk of multimorbidity (0.19).

**Conclusion:**

This study offers a comprehensive analysis of multimorbidity among older adults in India, revealing a risk burden driven by aging, poor childhood health, and socioeconomic disparities. By using an inclusive morbidity framework and CART modeling, it underscores the multifactorial nature of multimorbidity and highlights key interconnected determinants for future policy interventions.

## 1. Introduction

Low and middle-income countries (LMICs) are facing a rapid increase in disease burden from chronic diseases [1], primarily driven by the escalating aging population and changing lifestyle factors [2,3]. Coextensively, India is also passing through a parallel phase of demographic and epidemiological transition where diseases burden since the last decade is rapidly shifting from Communicable Diseases (CDs) to Non-communicable Diseases (NCDs) [4,5]. Since the early 2000s this epidemiological transition has been accompanied by sequential rising levels of multimorbidity, which the World Health Organization (WHO) defined as the coexistence of two or more morbidities or chronic conditions [6]. Multimorbidity is a complex and evolving public health challenge and associated with higher levels of healthcare service utilization and out of-pocket expenditures [7,8], elevated mortality risks [9–11], low quality of life  [ 12,13], complex treatments, and behavioural risk factors with psychological antecedents and consequences [9,14]. Multimorbidity also acts as an intermediate risk factor linking socioeconomic, demographic, and lifestyle factors to poor quality of life in later years among older adults in India [15]. Moreover, the recent Longitudinal Ageing Study of India (LASI, Wave 1, 2017−18), reported that nearly one-third of the population aged 45 and above are suffering from one of the five common chronic morbidities (hypertension, diabetes, cardiovascular diseases, chronic respiratory diseases, arthritis) [16]. Prior studies in India estimated the burden of multimorbidity to be over 50% among 45-year and above adults [17,18]. A recent study also showed that higher education, obesity, current employment, and poor childhood health were linked to earlier and more severe multimorbidity, especially among women [19] . This morbidity and thus multimorbidity burden in India is rapidly increasing due to increased unhealthy aging with longevity and early exposure to chronic disease risk factors [20]. 

Despite the notable rise in the prevalence of multimorbidity in recent decades, there remains a significant gap in understanding the conditional role of socioeconomic, demographic and lifestyle risk factors to multimorbidity among older adults in India. Although early studies identified risk factors for multimorbidity were aging, higher number of previous diseases, and lower education level, whereas a large social network seemed to play a protective role [21,22]. However, the approach of early studies in India for estimating the risk of multimorbidity primarily relied on odds ratios or relative risk models [20,23–25]. Thus, those models were not well-suited for capturing the non-linear relationships and the conditional role of background factors for multimorbidity susceptibility.

Moreover, if we try to understand the association of socioeconomic, demographic and lifestyle factors on multimorbidity susceptibility we get a labyrinth full of adjusted and unadjusted linear association. Thus, to unwind and understand this labyrinth of linear associations, it seems vital and essential to meticulously discover the nonlinear and conditional association with leading factors for multimorbidity susceptibility. The aim of this study is therefore to characterize and calculate the distribution, prevalence and relative share of multimorbidity prevalence burden over age and finally to identify the optimal leading covariates for multimorbidity and their condition role for multimorbidity susceptibility among older adults in India.

## 2. Materials and methods

### 2.1 Study population

LASI is a longitudinal survey (Wave 1, 2017−18) that provides data on many morbidities in individuals 45 years and above and their spouses irrespective of age and their socio-economic and demographic details, covering 28 states and 8 UTs of India. LASI has adopted a multi-stage stratified areas probability cluster sampling design, with three stages in the rural and four stages in the urban areas, respectively. Firstly, a primary sampling unit (PSU) was selected from each state/union territory (UT), followed by a village (from rural) or ward (from urban) area in the second stage. Finally, households were selected from the rural areas. However, in urban areas, census enumeration blocks (CEB) were selected randomly from each urban area, after which households were chosen from the selected CEB (International Institute for Population Sciences (IIPS), 2020). The present study utilised merged information from individual and biomarker datasets. The dataset contained samples of 73,396 adults aged 45 years and above, and in this research work, we primarily analysed data from 66,606 individuals after excluding the missing data.

### 2.2 Data availability and ethical consideration

Prior informed consent (written and verbal) from all the participants was collected by the field survey agencies. LASI administered consent forms at household and individual levels, following the Human Subject Protection [26]. The Indian Council of Medical Research (ICMR) extended the necessary guidance and mandatory ethical approval for conducting the LASI survey. The Institutional Review Board at IIPS provided additional approval for the study protocol. All methods were carried out under relevant guidelines and regulations by the ICMR.

### 2.3 Variables definitions

#### 2.3.1 Morbidities.

The study encompasses a total of thirty-six morbidities: Arthritis, Rheumatism, Osteoporosis, Bone joint other (Bone fracture, bone swelling, gout and haemophilia), Hypertension, Heart attack, Heart blockage, Heart failure, Arrhythmias, Heart rheumatic, Heart congenital or Structural disorders, Stroke, Heart other (Heart functioning, coronary thrombosis, myasthenia graves- post thymectomy and pericardial effusion), Asthma, Bronchitis, COPD (Chronic Obstructive Pulmonary Disease), Depression, Dementia, Neurological conditions, Psychiatric conditions, Cataract, Glaucoma, Hypermetropia, Myopia, Presbyopia, Diabetes and Thyroid, Jaundice or Hepatitis, Tuberculosis, Anaemia, Cancer, Cholesterol, Hearing conditions, Gastrointestinal conditions, Urogenital conditions and Skin diseases.

#### 2.3.2 Outcome variable.

All thirty-six morbidities and conditions were classified into binary forms (absent & present) and morbidities score were generated. Further, this score was categorised into three categories:

1) Zero morbidity (individuals with no morbidity or chronic health condition).
2) Single morbidity (individuals with only one morbidity or chronic health conditions}
3) Multimorbidity (individuals who had combinations of two or more morbidities or chronic health condition)

Morbidity information was based on self-reported data and documented diagnoses from the LASI dataset, obtained through participant responses to survey questions such as “Has any health professional ever diagnosed you with the following chronic conditions or diseases?” and “In the past two years, have you had any of the following diseases?”

#### 2.3.3 Exogenous factors.

We considered socioeconomic, demographic, lifestyle, and self-induced risk factors to explore the differentials in the prevalence of multimorbidity. Further, we adopted Anderson’s healthcare utilization framework [27] for the categorising of the covariates or exogenous factors in the group. In particular, the framework was suitable for characterizing the population at risk across three distinct subgroups, allowing for a comprehensive and systematic analysis of the factors influencing multimorbidity.

- Predisposing factors were Age, Sex (Male/Female), Caste group (SCs/STs, OBC, Others)2, religion (Hindu, Muslim, Christian & Others), Marital status (Currently Married, Widowed, Divorced/Separated/N), Living arrangement (Alone, Spouse, Other), Impairment (No/ Yes).
- Enabling factors were Level of education (No schooling, < 5 Years,5–9 years, 10 + years), MPCE quintiles (poorest, poorer, middle, richer, richest), Residence (Rural, Urban), Regions (North, Central, East, Northeast, West, South), Working status (never worked, currently working, currently not working), Childhood health (Very good, Good, Fair, Poor, Very Poor), Self-rated health (Good, Moderate, Poor), Physical activity (Everyday, Weekly, Casual).
- Self-induced and lifestyle risk factors were Tobacco consumption (Lifetime abstainer, Smokes tobacco, Smokeless tobacco, Both), Alcohol consumption (Lifetime abstainer, Infrequent non-heavy drinker, Frequent non-heavy drinker, Heavy episodic drinker) and BMI (Body Mass Index) as Underweight, Normal, Overweight, Obese. A comprehensive description of all exogenous factors (covariates) is available  in the S1 Table.

### 2.4 Statistical method

First, we computed the weighted prevalence distribution of morbidities and multimorbidity of the target population. Second, we plotted the relative proportional share of morbidities over age and calculated the weighted age-specific relative prevalence share of multimorbidity, single morbidity and zero morbidity. Third, we hypothesized that the distribution of Multimorbidity may be similar or different by the covariates; for this purpose, the Chi-square test was employed to examine differences in the distribution of multimorbidity across socioeconomic, demographic and lfestyle factors. Finally, the susceptibility to multimorbidity was assessed using the CART model, based on a set of dynamic exogenous determinants. Further, all p-values were two-tailed, and differences were considered statistically significant at p < 0.05.

#### 2.4.1 CART model.

The Classification and Regression Tree model (CART) is a method of regression or classification trees and used as a growing method for the decision tree, run for the one outcome variable (Multimorbidity, Single morbidity and Zero morbidity).

The CART model splits the data with maximum homogeneity within the node. The degree of a non-homogeneous subset in a node, is an indication of impurity. In selecting the best splitter, CART attempted to maximize the average purity of the two child nodes. The way to measures of purity can be chosen freely, can be called a splitting criterion, or splitting functions. For this study, this impurity is measured using the Gini index. Calculation of the Gini index was obtained by the below formula


$$\begin{array}{c} Gini(t)=1-\sum_{i=1}^{c-1}{\left[p\left(i|t\right)\right]}^{2} \end{array}$$

Where P (i | t) was the relative frequency of class i at node t, and c was the number of classes.

These calculations will achieve the highest value if the distribution of uniform class and has the smallest value if it contains all the records with the same class. Splitting of the data continues until homogeneity or stopping criteria are met in the node [28]. The minimum decrease in impurity was set at 0.0001. The growth limit was predetermined on a maximum tree depth of 5 and minimum leaf node observation of 5. Missing values were treated as missing values.

Three distinct strengths of CART that make it particularly applicable for analysing complex diseases models [29]. First, the hierarchical structure of CART models is often more intuitive than traditional regression models because it mimics the heuristics of decision making with conditional risks. Second, CART can outperform standard regression models when predicting outcomes in the presence of nonlinear relationships and interactions thresholds may vary with direct and intermediating risk factors. For example, the risk of diagnosis with CVDs in the individuals decreases if they have later onset of cholesterol and increases in the case of early onset of cholesterol. Third, CART affords the data greater freedom to speak for themselves. Whereas regression models are refined by comparing across a limited number of possible specifications, CART performs an exhaustive search over all possible cut-points and predictors [30]. As a result, the precise form of the relationship between a predictor and outcome is not delimited by the inclusion/exclusion of higher order terms.

All analyses were performed with SAS version 9.4.M8, R Studio version 2024.04.2−764 and STATA 17. A. The CART model was constructed using the “rpart” package in R and the resulting decision tree was visualized using the “rpart.plot” package.

## 3. Results

### 3.1 Prevalence, distribution and patterns of multimorbidity and morbidities

Table 1 shows the burden of morbidities among the older adult population aged 45 and above in India. Zero morbidity prevalence was 25.29%, single morbidity prevalence was 23.78%, and multimorbidity was 50.90%. Amongst 36 diseases, hypertension showed the highest burden of 26.72%, closely followed by myopia (24.2%), and bone-joint & other diseases (0.01%) had the lowest prevalence. Most chronic morbidities showed prevalence in the range of 1–7%, with a mean value of 5%. Therefore, morbidity with a high prevalence of hypertension, myopia, hypermetropia (20.75%), gastrointestinal condition (17.98%), cataracts (14.18%), and diabetes (12.21%) confirmed a larger morbidity burden in the Indian population compared to other morbidities. It indicates the predominance of few acute and degenerative diseases for, unhealthy aging among older adults in India.

**Table 1:** Prevalence distribution of causes and thirty-six morbidities and conditions among older adults, LASI Wave-1 (2017−18), India.

Causes	Weighted Frequency(N = 66,606)	Weighted Percentage % (95% CI)
Zero Morbidity	16842	25.29 (24.96-25.62)
Single Morbidity	15837	23.78 (23.45-24.10)
Multimorbidity	33927	50.94 (50.56-51.32)
**Morbidities and Conditions**		
Hypertension	17796	26.72 (26.38-27.06)
Myopia	16090	24.16 (23.83-24.48)
Hypermetropia	13823	20.75 (20.45–21.06)
Gastrointestinal conditions	11978	17.98 (17.69–18.28)
Cataract	9448	14.18 (13.92–14.45)
Diabetes	8130	12.21 (11.96–12.46)
Arthritis	5978	8.97 (8.76–9.19)
Hearing conditions	4593	6.90 (6.70–7.09)
Presbyopia	4595	6.90 (6.71–7.09)
Urogenital conditions	4303	6.46 (6.27–6.65)
Rheumatism	4100	6.16 (5.97–6.34)
Skin diseases	3475	5.22 (5.05–5.39)
Asthma	3096	4.65 (4.49–4.81)
Anaemia	3061	4.60 (4.44–4.76)
Heart attack	2505	3.76 (3.62–3.91)
Osteoporosis	2213	3.32 (3.19–3.46)
Thyroid	1871	2.81 (2.68–2.94)
Jaundice or Hepatitis	1858	2.79 (2.67–2.92)
Cholesterol	1484	2.23 (2.12–2.34)
Glaucoma	1264	1.90 (1.80–2.00)
Stroke	1262	1.89 (1.79–2.00)
Neurological conditions	915	1.37 (1.29–1.47)
COPD	902	1.35 (1.27–1.44)
Bronchitis	798	1.20 (1.12–1.28)
Arrhythmias heart	746	1.12 (1.04–1.20)
Tuberculosis	681	1.02 (0.95–1.10)
Heart blockage	671	1.01 (0.93–1.09)
Rheumatic heart	558	0.84 (0.77–0.91)
Heart failure	512	0.77 (0.70–0.84)
Cancer	425	0.64 (0.58–0.70)
Depression	402	0.60 (0.55–0.67)
Dementia	388	0.58 (0.53–0.64)
Psychiatric conditions	251	0.38 (0.33–0.43)
Heart Other^(a)^	116	0.17 (0.14–0.21)
Heart congenital or structural disorders	65	0.10 (0.08–0.12)
Bone joint other^(b)^	5	0.01 (0.00–0.02)

^a^ *– Heart functioning, coronary thrombosis, myasthenia graves- post thymectomy and pericardial effusion.*

^b^ *– Bone fracture, bone swelling, gout and haemophilia.*

**Fig 1:**
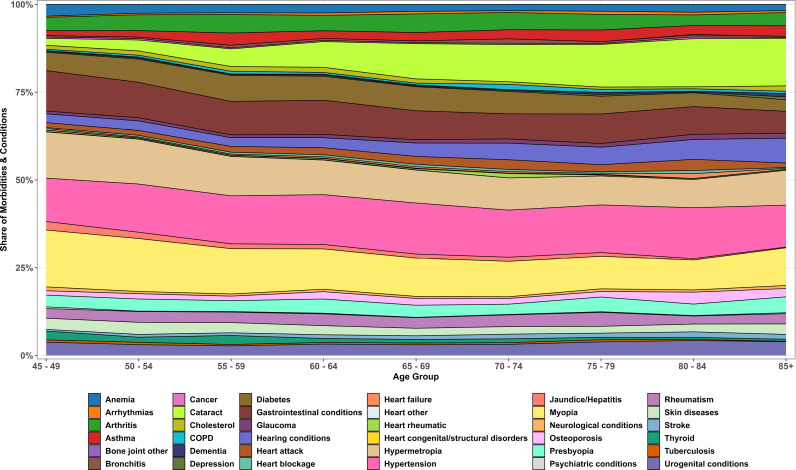
Age-specific relative share of thirty-six morbidities and conditions, LASI Wave-1 (2017−18), India.

Fig 1 illustrates the relative age-specific distribution of morbidity composition among adults aged 45 years and above. The stacked area chart reveals that a limited number of non-communicable diseases, particularly hypertension, diabetes, and cataract, constitute a substantial proportion of the total morbidity burden across all age groups. While the relative contribution of hypertension and diabetes slightly declines in older age cohorts, conditions such as cataract and hearing disorders become more prominent, reflecting age-associated degenerative changes. Less prevalent morbidities, including congenital, psychiatric, and skin-related conditions, consistently contribute minimally across the lifespan. However, the relative share of severe diareses like tuberculosis, cancer and heart diseases in all groups remained significantly small and constant across all age groups.

**Fig 2:**
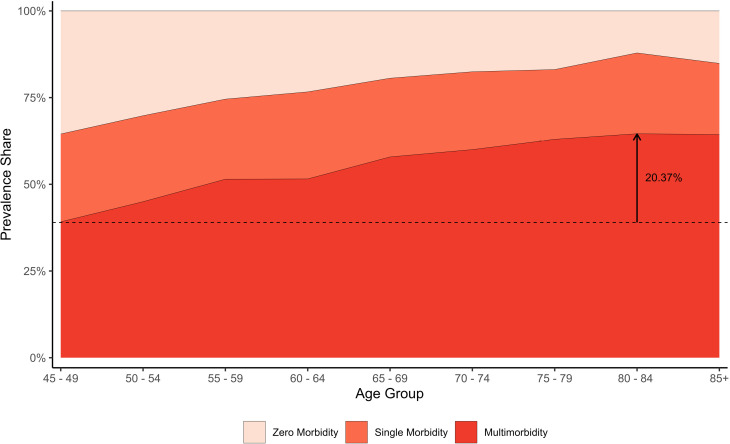
Age-specific relative proportional distribution of Zero morbidity, Single morbidity and Multimorbidity, LASI Wave-1 (2017−18), India.

Fig 2 depicts the relative prevalence share of Multimorbidity, Single morbidity and Zero morbidity over the age groups. The share of multimorbidity expanded by 20.37% when comparing individuals aged 45–49 (43.47%) to those aged 80–84 years (63.84%), with relative percentage change of 46.87. This indicates that as the population ages, particularly into the oldest age group, they are significantly more likely to experience multiple chronic conditions. On the contrary single morbidity proportion remained constant and the relative share remained plateaued across all age groups, while zero morbidity share shirked in later ages, this reduction was largely offset by a corresponding rise in the burden of multimorbidity in later age-groups among the older adults in India.

**Fig 3:**
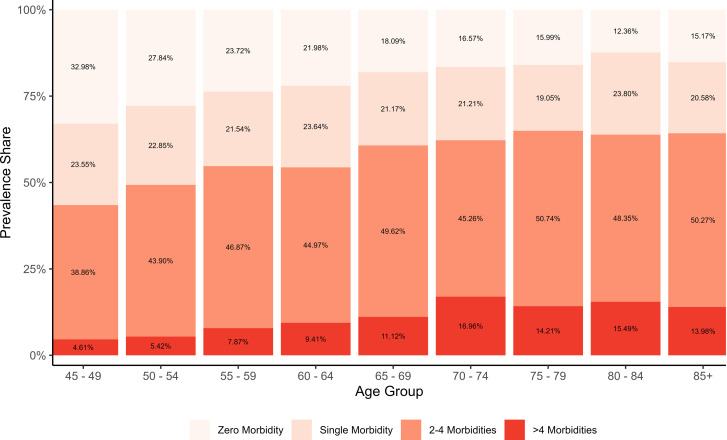
Age-specific relative proportional distribution of multimorbidity counts, LASI Wave-1 (2017−18), India.

Further, in Fig 3,the stacked bar plot illustrates a pronounced age-related shift   within the multimorbidity burden among older adults in India. The prevalence of zero morbidity steadily declines from 32.98% at ages 45–49 to 15.17% among those aged 85+, reflecting a reduction of over 17 percentage points and indicating diminishing health with advancing age. Single morbidity shows a modest decline from 23.55% to 20.58%, suggesting a shift toward more complex health conditions in older age. In contrast, the proportion of individuals with 2–4 morbidities increase consistently from 38.86% in the 45–49 group to 50.74% in the 75–79 group, followed by a slight decline to 50.27% in the 85+ group, suggesting a high but plateauing burden in the oldest-old. Similarly, >4 morbidities rise markedly from 4.61% at ages 45–49 to a peak of 16.96% in the 70–74 group, before declining slightly to 13.98% at 85+. These patterns reveal that the overall burden of multimorbidity intensifies with age, predominantly driven by individuals with 2–4 morbidities; however, a notably steep increase is also observed in the prevalence of >4 morbidities, particularly up to the mid-70s. Interestingly, a modest decline or plateau in the oldest age groups suggests the potential influence of survivorship bias or selective mortality, wherein individuals with the highest disease burden may not survive into advanced old age.

Table 2 presents the weighted percent distribution of the three categories of morbidity status, by background characteristics in the Indian older adults population aged 45 years and above. The background variables were categorised as predisposing, enabling, and risk factors. Among the predisposing factors, women exhibited a slightly higher prevalence of multimorbidity (51.86%) compared to men (49.85%), indicating a marginal gender disparity in disease burden. By place of residence, multimorbidity was 62.99% in the urban population compared to 45.4% in the rural population. Social groups are of differential importance in Indian society; among social groups, the upper caste category showed a large share of 60.19% in the multimorbidity category. On the other hand, the most socially vulnerable STs category showed a small share of 31.12% in the multimorbidity category. It indicates a lack of medical infrastructure can be a factor for assessing medicine and drug facilities as when required in a society.

**Table 2:** Descriptive characteristics of Zero morbidity, Single morbidity and Multimorbidity by exogenous facors, LASI Wave-1 (2017−18), India.

Covariates	Zero Morbidity	Single Morbidity	Multimorbidity	Frequency	Weighted Percentage	P – Value
**Sex**						
Male	25.94	24.21	49.85	30600	45.94	< 0.001
Female	24.73	23.41	51.86	36006	54.06	
**Residence**						
Rural	28.57	26.00	45.40	45648	68.53	< 0.001
Urban	18.13	18.88	62.99	20958	31.47	
**MPCE quintile** ^(a)^						
Poorest	32.88	27.25	39.87	13896	20.86	< 0.001
Poorer	26.65	24.72	48.64	14144	21.24	
Middle	26.24	24.00	49.76	13646	20.49	
Richer	21.99	22.53	55.48	12937	19.42	
Richest	17.34	19.73	62.93	11984	17.99	
**Highest level of Schooling**						
No schooling	29.69	25.82	44.49	33722	50.63	< 0.001
< 5 Years	21.95	22.37	55.68	7266	10.91	
5 - 9 Years	21.01	23.17	55.82	13594	20.41	
10 + Years	19.79	19.58	60.63	12023	18.05	
**Religion**						
Hindu	25.95	24.35	49.71	54590	81.96	< 0.001
Muslim	22.03	21.21	56.77	7667	11.51	
Christian	30.81	21.48	47.71	2023	3.04	
Other	15.71	20.88	63.42	2327	3.49	
**Caste Category** ^(b)^						
ST	41.93	26.95	31.12	5732	8.61	< 0.001
SC	26.66	25.17	48.00	12759	19.16	
OBC	25.57	24.02	50.40	30272	45.45	
Other	18.47	21.34	60.19	17843	26.79	
**Working Status**						
Never worked	22.35	21.90	55.75	17357	26.09	< 0.001
Currently working	30.70	25.47	43.82	33248	50.00	
Currently not working	17.13	22.31	60.56	15914	23.92	
**Current Marital Status**						
Currently married	26.50	24.16	49.34	48855	73.35	< 0.001
Widowed	20.34	22.62	57.03	15799	23.72	
Divorced/separated/other	34.93	23.52	41.56	1952	2.93	
**Region**						
North	21.36	22.94	55.70	8170	12.27	< 0.001
Central	35.84	27.17	36.99	13699	20.57	
East	24.91	25.24	49.85	15420	23.15	
Northeast	30.92	25.09	44.00	2320	3.48	
West	18.43	20.96	60.60	10990	16.50	
South	22.51	21.63	55.86	16008	24.03	
**Alcohol Consumption**						
Lifetime abstainer	24.40	23.60	52.00	55369	89.3	< 0.001
Infrequent non-heavy drinker	22.34	22.98	54.69	4146	6.69	
Frequent non-heavy drinker	29.24	26.31	44.45	1601	2.58	
Heavy episodic drinker	33.32	26.92	39.76	885	1.43	
**Tabacco Consumption**						
Lifetime abstainer	24.41	22.71	52.89	42108	63.22	< 0.001
Smokes tobacco	26.74	24.85	48.41	9273	13.92	
Smokeless tobacco	27.24	25.81	46.95	13243	19.88	
Both	24.11	27.93	47.96	1982	3.00	
**SRH** ^(c)^						
Good	34.36	27.27	38.37	24735	37.66	< 0.001
Moderate	22.98	23.31	53.72	28864	43.94	
Poor	11.29	18.49	70.22	12089	18.40	
**Childhood Health** ^(d)^						
Very good	24.05	24.21	51.74	31875	48.56	< 0.001
Good	25.94	24.10	49.96	25535	38.90	
Fair	27.38	21.91	50.72	7205	10.98	
Poor	22.73	21.71	55.56	943	1.44	
Very Poor	10.79	13.86	75.34	84	0.13	
**BMI** ^(e)^						
Underweight	32.28	28.11	39.61	12815	21.39	< 0.001
Normal	26.83	24.97	48.21	30870	51.52	
Overweight	17.29	20.93	61.79	12152	20.28	
Obese	11.37	17.11	71.52	4081	6.81	
**Living Arrangement**						
Alone	21.24	22.57	56.19	2454	3.68	< 0.001
Spouse	26.51	24.14	49.35	48179	72.33	
Others	22.21	22.88	54.91	15973	23.98	
**Physical Activity**						
Everyday	31.09	26.01	42.90	16634	25.19	< 0.001
Weekly	30.48	25.83	43.69	6746	10.22	
Casual	21.71	22.77	55.53	42647	64.59	
**Impairment** ^(f)^						
No	26.15	24.13	49.71	61082	91.72	< 0.001
Yes	15.68	19.83	64.49	5516	8.28	

(a) *– Wealth index (Monthly per capita consumption expenditure);* ^(b)^ *– SC – Scheduled Castes, ST – Scheduled Tribes, OBC – Other Backward Classes;* ^(c)^ *– Self Rated health;* ^(d)^ *–* *In general, what was the overall childhood health of individual up to age 16 years;* ^(e)^ **–* Body mass index, Underweight (BMI ≤ 18.4 kg/m*^*2*^*), Normal (18.5 kg/m*^*2*^* ≤ BMI ≤ 24.9 kg/m*^*2*^*), Overweight (25.0 kg/m*^*2*^* ≤ BMI ≤ 29.9 kg/m*^*2*^*), Obese (BMI ≥ 30 kg/m*^*2*^*);* ^(f)^ **–* Any form of physical or mental impairment*

Amongst religions, Hindus with the largest base showed a share of 49.71%, Christians with the smallest sample size showed a share of 47.71%, and others with a small sample size showed the largest share of 63.42% in the multimorbidity category. By marital status, the share of multimorbidity was 57.03% in widowed, followed by a share of 49.34% in the currently married person category. The share of multimorbidity was higher in persons living alone (56.18%) than in persons living with spouses (49.35%). Also, persons living with disability or impairment show a larger share in the multimorbidity category compared to normal persons. The predisposing factors explain a large share of the Indian population in the multimorbidity category, except in the young-adult age group of 45–49 years.

Amongst enabling factors, the share of multimorbidity was 60.63% in persons with schooling for more than ten years versus 44.49% in persons without schooling. As measured by the MPCE class, the affluent class showed a large share of 62.93% in the multimorbidity category, whereas the poorest class showed a share of 39.87%. By work status, persons currently not working showed a larger share of 60.56% in the multimorbidity category compared to those currently working with 43.82%.

Childhood health has long-term implications in the later years of an individual’s life. Individuals who had very poor childhood health endured a large share of 75.34% in the multimorbidity category in comparison to individuals who had better childhood health showing a share of ~50%. Amongst better childhood health conditions, persons with very good childhood health statuses showed a share of 51.74% in multimorbidity and the remaining 24.21% in single morbidity and 24.05% in zero morbidity. It implies that serious illness in childhood is the early origin of multimorbidity. A similar distribution of morbidity status was also apparent for self-rated health status. Individuals with poor self-rated health status reported a large share of 70.22% in the multimorbidity category compared to persons with good self-rated health status showing 38.37%. It points out an affirmative relationship between low self-rated health status and the prevalence of multimorbidity in the population. By region, the western, northern, and southern regions showed a larger percentage of the population enduring multimorbidity compared to the eastern, central, and north-eastern regions. Regional diversity in multimorbidity is expected as many states of India experience variations in weather and monsoons, which affects the level of mortality and morbidity. Most of these enabling factors reveal a large proportion of multimorbidity in populations with lower levels of health conditions and lower socio-economic status compared to their counterparts.

A large percentage of the obese (71.52%) and overweight (61.79%) population had multimorbidity; contrarily, a small percentage of the underweight population (39.61%), another facet of malnutrition, had multimorbidity. Finally, by applying the chi-square test of independence concludes a statistically significant association between the outcome variable (morbidity status) and covariates, as the P-value at the 5% level of significance was less than 0.001 for each covariate.

### 3.2 Conditional role of exogenous factors on multimorbidity susceptibility

**Fig 4:**
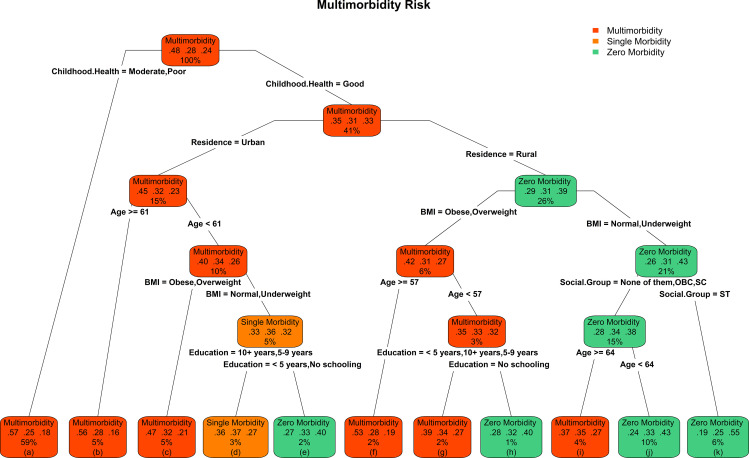
CART tree with hierarchical condition risk for multimorbidity susceptibility by exogenous factors, LASI Wave-1 (2017−18), India.

To further identify the optimal leading covariates and their joint conditional role for multimorbidity susceptibility among older adults in India, we constructed the CART model. Fig 4 depicts the conditional risk and sample size of each of the three outcomes in each box respectively: Zero morbidity (right digit), Single morbidity (middle digit) and Multimorbidity (left digit). CART model deducted that childhood health, place of residence, age, BMI, caste category and education level are the leading optimal covariates (Table 3) for multimorbidity risk among older adults in India.

**Table 3:** Leading factors of multimorbidity susceptibility, LASI Wave-1 (2017−18), India.

Leading Covariates	Standardized Variable Importance (VIMP)
Childhood health	10.50
Residence	2.85
Age	2.26
BMI	1.99
Caste category	1.36
Highest level of schooling	0.83

The leftist terminal node (a) showed the highest risk of multimorbidity (0.57) was among those individuals who had moderate or poor childhood health. This risk probability is followed by the insignificant differential of risk (0.56) among those individuals whose age > 60 (b), who resides in urban areas and had good childhood health. While this risk shifted to 0.47 in addition to above leaf nodes among those individuals who were obese or overweight and were less than 61 years old (c). Conversely, individuals who were ST, had normal or underweight BMI and resided in rural (k) were at least risk of multimorbidity (0.19). In the same arm of BMI, a similar risk pattern (0.24) was also observed among those individuals whose age was less than 64 and who didn’t belong to the ST group (j). However, multimorbidity risk was elevated manifold for those who were more than 63 years of age (i). Level of education played a vital significant role in both single and multimorbidity risk. The risk of single morbidity was highest (0.37) among those individuals who had 10 plus years of education, had normal or underweight BMI, resided in rural and had good childhood health (d). However, this risk shrined to (0.33) for those who had no schooling or less than 5 years of schooling (e). Parallelly for multimorbidity, this risk shifted from 0.36 to 0.27.

A distinctive pattern in conditional risk shift was observed for multimorbidity among rural residents. The risk of multimorbidity is 0.53 among those Individuals whose age was greater than 56, obese or overweight, who resided in rural and who had good childhood health (f). Moreover, this risk decreases to 0.39 for those individuals whose age was less than 57 and who had education (g). However, this risk further decreases to 0.28 for those individuals who had no schooling (h).

The CART model achieved an overall classification accuracy of 88%, with a kappa statistic of 0.78, indicating substantial agreement. The model demonstrated strong sensitivity and specificity across all multimorbidity categories. A pseudo R-squared of 0.56 and a low cross-validation error indicate good model fit and reliable predictive performance.

## 4. Discussion

In this study, we used the comprehensive nationally representative data of India and extensively explored the relative distribution, pattern and conditional role of risk factors for multimorbidity susceptibility among older adults in India. Additionally, this study had broad inclusion criteria of NCDs, CDs and endemic morbidities to provide both an in-depth and comprehensive understanding of morbidities and multimorbidity. The overall findings highlighted that over the aging period, the spectrum of multimorbidity and acute morbidities burden had undergone a dramatic transition from middle to oldest of old age groups, coupled with the sequential multiplicity of multimorbidity burden in the oldest of old age groups. Moreover, in this paper the highest multimorbidity susceptibility risk was frequently paired with early poor childhood health along with conditional role of nonmodifiable risk factors such as age and sex.

This study has several new key findings. First, morbidities prevalence was consistent with other parallel studies [31–33], while the multimorbidity prevalence (50.94%) estimate was significantly reached to its threshold in this study is contrary to previous studies in India which ranges from 7.2% to 45.3% [20,34–37]. The plausible reason for such a huge burden is due to an extensive number of NCDs, CDs and endemic morbidities were included in this study, where degenerative and acute morbidities, which can go on for years, captured the major share of the disease burden over all age groups among older adults in India.

Moreover, it is essential to note that in the broad spectrum of morbidity burden among older adults in India, eye disorders and gastrointestinal conditions showed a predominance burden, however with the lowest fatality, hypertension had the highest burden of 26.72% across all age groups, indicating a salient role of either mediator, moderator or confounder. Nonetheless, recent studies complemented these findings by indicating the role of hypertension as the underlying risk factor for other morbidities [38–41]. Furtermore, the burden of chronic and communicable disease was significantly low, but these patterns were substantiated by coherent of high mortality patterns [37]. Among chronic morbidities, asthma and heart attack had the highest burden, while cancer with the highest mortality risk had a prevalence of only 0.64%. Additionally, we also complemented this study analysis by showing how the disease burden profile transitions over later ages followed by the sequential expanding share of multimorbidity burden by 25.2% from older to oldest of old age groups. On the dissident, the single morbidity proportion remained constant and the relative share remained plateaued across all age groups, while zero morbidity share shirked in later ages.

In addition to these findings, this paper extended evidence on the role of socioeconomic, demographic, modifiable and unmodifiable risk factors in multimorbidity composition. The population of the oldest of the old group experiences disproportionality higher rates of multimorbidity, while older groups show more balanced distributions between multimorbidity and good health. In line with the earlier studies, gender differences are subtle but present, while urban individuals consistently exhibit higher prevalence than their rural counterparts [20,31,37]. Higher wealth quintile and education levels surprisingly correlate with increased rates, possibly due to better living, diagnostic access or lifestyle factors. Lower socioeconomic groups, particularly Scheduled Tribes, show lower rates, potentially indicating underreporting or barriers to healthcare. Religious and regional variations suggest cultural and geographical influences, with some groups, like Hindus and non-SC or ST caste, showing higher rates [20,42]. Congruent with former studies in India, self-induced substance abuse like smoking and alcohol consumption, also contributes significantly to the multimorbidity share [43,44]. We extended the previous studies’ findings that the place of residence and age play a very significant factor in multimorbidity risk [20,44]. Furthermore, there were few parallel collateral effects, including the education level of the individuals, which include higher education levels associated with higher multimorbidity risk, particularly in urban areas. These findings collectively underscore the complexity of multimorbidity, driven by a combination of biological, social, and environmental factors.

Finally, with the contention that multimorbidity risk is not solely driven by isolated factors, this study used the comprehensive CART model to provide a fundamental and comprehensive baseline understanding for future studies by examining the underlying multimorbidity susceptibility within the context of vital conditional risk factors among older adults in India. A systematic review synthesizes and identified age, gender, marital status, economic dependence, and smoking as key conventional risk factors for multimorbidity in India [44]. In contrast, our study highlights childhood health, place of residence, age, BMI, caste category, and education level as the primary conditional determinants of multimorbidity susceptibility for the older population, suggesting a broader set of influences beyond traditionally recognized factors [44]. Surprisingly in contrary to the previous studies, behavioural and self-induced risk factors like smoking and alcohol consumption did not emerge as the leading contributors to multimorbidity risk, highlighting the limitation of confounding or mediating effects in prior studies [43,45,46]. Moreover, a recent study in India and Brazil highlighted that those individuals who perceived their childhood health as poor had the highest level of multimorbidity with (APR: (India: 1.38, 1.16 to 1.65) and (Brazil: 1.19, 1.09 to 1.30) [47] and indicated a clear association between aging and the accumulation of conditions. Our study’s outcomes hold important discussion for the evolving narrative surrounding the individual’s childhood health, as the highest risk of multimorbidity suspensibility was observed among those individuals who had poor childhood health and this pattern was closely followed by those over 60 years old living in urban areas with good childhood health (0.56). Conversely, the lowest risk of 0.19 was among rural residents from Scheduled Tribes (ST) with normal or underweight BMI, suggesting that BMI plays an important role in vulnerable populations. 

This study has a few limitations, although our analysis and approach were novel, as it integrates evidence from robust and new statistical methods with a unique approach. Our study included a more comprehensive list of morbidities that has not been considered in previous studies of multimorbidity in India, yet it was not possible to include function limitation, Activities of daily living (ADL) and Instrumental activities of daily living (IADL) variables as these identified as confounders. Moreover, 45 years and above adults are considered in our study, which may have reduced variability and potentially increase the difficulty of identifying an association between factors and multimorbidity. A notable limitation of the study is that multimorbidity is treated as a general burden of chronic conditions without accounting for specific disease clusters. The CART model identifies patterns and conditional associations but does not establish causal relationships. Observed risk factors may be influenced by data structure, confounding effects, or hidden biases rather than true causal effects. Future studies should complement this approach with causal inference methods for deeper insights. Finally, a notable limitation of the study is that multimorbidity is treated as a general burden of chronic conditions without accounting for specific disease clusters. It is well recognized and established fact that certain groups of conditions (e.g., CVDs, heart diseases, neuropsychiatric disorders) may share common risk pathways due to underlying causal mechanisms.

### 4.1 Conclusion

In conclusion, this study for the first time incorporated thirty-six morbidities for evaluating the composition, multiplicity and examines the underlying multimorbidity susceptibility within the context of conditional risk factors among older adults in India. Nonetheless, this study also specifies the relative share of multimorbidity burden over age. The study highlighted that acute and degenerative morbidities had a huge burden across all age groups, while complex multimorbidity share expanded significantly in the oldest of old age groups. Furthermore, our results are complemented with extended evidence on the role of socioeconomic, demographic and modifiable or unmodifiable risk factors in multimorbidity composition. Childhood health was the key leading determinant of multimorbidity susceptibility and enhanced our understanding of healthy aging and place of residence, age, BMI and caste category were identified as the leading key factors for multimorbidity susceptibility among older adults in India. Finally, to address the significant issue of multimorbidity among older adults in India, it is essential to implement integrated health programs that focus on both NCDs and CDs through holistic care models in primary healthcare. Initiatives should include preventive health education targeting childhood health, socioeconomic support for vulnerable groups, and health literacy campaigns that promote healthy lifestyles.

## Supporting information

S1 Table Detailed descriptions of the covariates, Longitudinal Ageing Study in India (LASI), wave-1, 2017–2018. (DOCX)
