# Supplementary material for: Towards a new perspective: Exploring the variability of conditional risk factors for multimorbidity susceptibility among older adults in India
Source: PLoS One. 2025 Jun 17;20(6):e0323890. doi: 10.1371/journal.pone.0323890 (PMC12173406; doi:10.1371/journal.pone.0323890)
Supplement: S1 Table — (DOCX) [file pone.0323890.s001.docx]

**S1 Table: Detailed descriptions of all the covariates, Longitudinal Ageing Study in India (LASI), wave-1, 2017–2018**

| **Exogenous factors** | **Descriptions** |
| --- | --- |
| Age Group | Current age group of the individual |
| Sex | Sex of the individual |
| Residence | Place of current residence of the individual |
| MPCE quintile | MPCE - Monthly per capita consumption expenditure quintiles |
| Highest level of Schooling | Highest level of education that individual completed |
| Religion | Religion of individual |
| Caste Category | SC - Scheduled Castes  ST - Scheduled Tribes  OBC - Other Backward Classes |
| Working Status | Working status of the individual |
| Current Marital Status | Current marital status of individual |
| Region | Region that individual currently reside in |
| Alcohol Consumption | Lifetime abstainer - Individual never consumed any alcoholic beverages such as beer, wine, liquor, country liquor.  Infrequent non-heavy drinker - In the past three months, on an average had less than once a month alcoholic drink.  Frequent non-heavy drinker - In the last 3 months, less than once a month on average, individual had at least 5 or more drinks on one occasion.  Heavy episodic drinker - In the last 3 months, one to four days per week on average, individual had at least 5 or more drinks on one occasion. |
| Tabacco Consumption | Lifetime abstainer and Current user of Smokes tobacco, Smokeless tobacco, Both |
| Self-Rated Health (SRH) | Overall, general health of the individual (Good, Moderate, Poor) |
| Childhood Health | In general, what was the overall childhood health of individual up to age 16 years. |
| BMI | Body mass index as Underweight (BMI≤ 18.4 kg/m2), Normal (18.5 kg/m2 ≤ BMI ≤ 24.9 kg/m2), Overweight (25.0 kg/m2 ≤ BMI ≤ 29.9 kg/m2), Obese (BMI ≥ 30 kg/m2) |
| Living Arrangement | Current status of living arrangement (Alone, Spouse, Others) |
| Physical Activity | The type of physical activity included in the individual’s lifestyle. |
| Impairment | Does individual had impairment or health problem that limits the kind or amount of paid work can do. (Yes, No) |
